# Exposure to Endocrine Disruptors in Early life and Neuroimaging Findings in Childhood and Adolescence: a Scoping Review

**DOI:** 10.1007/s40572-024-00457-4

**Published:** 2024-07-30

**Authors:** Kim N. Cajachagua-Torres, Hugo G. Quezada-Pinedo, Tong Wu, Leonardo Trasande, Akhgar Ghassabian

**Affiliations:** 1 Department of Pediatrics, NYU Grossman School of Medicine, 555 First Avenue, New York, NY 10016 USA; 2 Department of Pediatrics, Erasmus MC, Erasmus University Rotterdam, Rotterdam, The Netherlands; 3 Department of Radiology and Nuclear Medicine, Erasmus MC, Erasmus University Rotterdam, Rotterdam, The Netherlands; 4 Department of Population Health, NYU Grossman School of Medicine, New York, NY USA

**Keywords:** Endocrine disruptors, Brain, Neuroimaging, Epidemiological studies

## Abstract

**Purpose of Review:**

Evidence suggests neurotoxicity of endocrine disrupting chemicals (EDCs) during sensitive periods of development. We present an overview of pediatric population neuroimaging studies that examined brain influences of EDC exposure during prenatal period and childhood.

**Recent Findings:**

We found 46 studies that used magnetic resonance imaging (MRI) to examine brain influences of EDCs. These studies showed associations of prenatal exposure to phthalates, organophosphate pesticides (OPs), polyaromatic hydrocarbons and persistent organic pollutants with global and regional brain structural alterations. Few studies suggested alteration in functional MRI associated with prenatal OP exposure. However, studies on other groups of EDCs, such as bisphenols, and those that examined childhood exposure were less conclusive.

**Summary:**

These findings underscore the potential profound and lasting effects of prenatal EDC exposure on brain development, emphasizing the need for better regulation and strategies to reduce exposure and mitigate impacts. More studies are needed to examine the influence of postnatal exposure to EDC on brain imaging.

**Supplementary Information:**

The online version contains supplementary material available at 10.1007/s40572-024-00457-4.

## Introduction

The ubiquitous exposure to endocrine-disrupting chemicals (EDCs) is an environmental health issue worldwide. EDCs are considered exogenous substance or mixture that interferes with hormonal regulation by mimicking hormones and subsequently disrupting endocrine functioning in humans or its progeny. As pregnancy is a vulnerable period for fetal development orchestrated by endocrine factors, prenatal exposure to EDCs may alter the embryos and fetal development by shifting the regulation of the maternal-placental-fetal triad.

Extensive evidence suggests that prenatal exposure to EDCs has been associated with alterations in fetal development and growth—even at low exposure levels [1–6]. EDCs can cross both the placental and blood–brain barriers, and thus impact brain development. Additionally, a growing body of literature shows that preterm birth, small for gestational age, and alteration in the fetal growth are linked to adverse cognitive and brain development [7]. Brain development begins early in gestation, followed by myelination in the second trimester, and both white matter development and myelination continue throughout childhood, adolescence, and young adulthood [8–10]. This requires complex maturational process including neurogenesis, neuronal migration, myelination and pruning [10]. Since many brain processes are responsive to tightly regulated hormonal function; endocrine disruption caused by exogenous sources can interfere with each one of the brain processes during prenatal, early childhood and adolescence period and subsequently influence neurobehavioral and cognitive outcomes [11–15].

Several observational studies have reported that higher concentration of EDCs during pregnancy are associated with changes in the fetal head circumference, biparietal diameter, and occipitofrontal diameter as measured by ultrasound [6, 16, 17]. Additionally, sex differences have been reported in animals and humans regarding EDC exposure and brain development [18]. Advances in neuroimaging, particularly in magnetic resonance imaging (MRI), and its application in population-based epidemiological settings have made it possible to identify and unravel brain influences of environmental exposures. This review aims to provide an overview of the available scientific information that links early life exposure to EDCs and brain development (structure and/or function) using different MRI modalities. We included studies that examined EDC exposure using biomonitoring as well as studies on air pollution, if included exposure to polyaromatic hydrocarbon (PAHs) and metals, as they have endocrine disrupting properties [19].

## Methods

### Search Strategy

We conducted a comprehensive literature search strategy using the PubMed and PsycINFO until January 11, 2024. The search identified 657 citations. Following initial screening of titles and abstracts, the full texts of 54 articles were evaluated further. Studies were included if they (a) were nested-case control, case–control, cross-sectional, prospective and retrospective cohort studies, and/or randomized clinical trial; (b) had reported the influence of at least one EDCs, including bisphenols, phthalates, perchlorate, organophosphates pesticides, Atrazine, DDT, 2,4-D, Glyphosate, perfluoroalkyl substances (PFASs), PAHs, heavy metals, phytoestrogens, polybrominated diphenyl ethers (PBDE), polychlorinated biphenyls (PCBs), dioxins, and triclosan—*from conception until 21 years old*; (c) had assessed the association with brain structure and/or function using neuroimaging—*from prenatal period until 21 years old*, and (d) were conducted in humans. We only included MRI studies, i.e., structural MRI, Diffusion Tensor Imaging (DTI), and functional MRI (fMRI). Brain structural MRI enables the measurement of various brain regions in volume, as well as area, surface, and thickness of cortical regions. DTI is focused on mapping the tracts and connectivity in the white matter. fMRI measures the changes in blood flow and oxygenation in the brain as response to neuronal connectivity. We excluded studies that used ultrasound. Finally, 46 articles were included, further details on the review process are described in Supplementary Material.

## Results

Table 1 and 2 present the overview of 46 studies according to the period of exposure. For each exposure period, we review studies according to the environmental compounds.

**Table 1:** Prenatal EDC and brain structure and/or function

Study ID	Study Design	Continent	Country	Sample size	Number of girls	Number of boys	Exposure	Source of exposure	Time of exposure	Neuroimaging modality	Age outcome	Findings	Sex differences	Adjustment for SES^#^
Zheng 2022	Cohort	North America	Canada	87	47	40	BPA	urine	T2	MRI	2.7–7.2 years (4.3 ±1.1)	**Volumes - small effect:**↓ opercular region of the inferior frontal gyrus, superior occipital gyrus, and postcentral gyrus volume.Structures in limbic system: ↑ hippocampus volume ↑ entorhinal area ↑ amygdala volume ↓ accumbens area **Hemispheric differences (left vs. right)** ↓ left superior occipital gyrus volume ↑ left postcentral gyrus volume ↑ right transverse temporal gyrus, medial frontal cortex, parahippocampal gyrus, ↓ right opercular part of the inferior frontal gyrus and frontal pole	**males—volume** ↑ gyrus rectus ↑ putamen ↑ medial frontal cortex ↓ opercular part if the inferior frontal gyrus **females- volume** ↓ superior parietal lobule ↓ planum polare ↓ frontal operculum ↓ medial orbital gyrus ↑ transverse temporal gyrus ↓ superior frontal gyrus medial segment	no
Grohs 2019	Cohort	North America	Canada	98	51	47	BPA	urine	T2,	DTI	3.73 ± 0.84 years	↑ MD in CC splenium and right IFL **Mediation analysis**: Splenium diffusivity mediated: BPA levels and children’s internalizing behavior	NA	no
Ghassabian 2023	Cohort	Europe	Netherlands	775	385	390	Phthalates	urine	T1,T2,T3	MRI	10 years	mEP (average): ↓ total gray matter volume **Mediation analysis:** Total gray matter volumes partially mediated: mEP and ↓ child IQ	**Only girls:** mIBP: ↓ cerebral white matter volumes cerebral white matter volumes mediated: mIBP and ↓ IQ	no
England-Mason 2020	Cohort	North America	Canada	76	39	37	Phthalates	urine	T2	DTI-MRI	3–5 years	HMWP/LMWP: ↓ FA in the left ILF ↑ MD in the right IFO, right ILF, right pyramidal fibers, left and right UF **Mediation analysis:** MD of the right IFO mediated: HMWP was indirectly associated with internalizing and externalizing problems MD of the right pyramidal fibers mediated: HMWP and internalizing problems	NA	no
Shen 2021	Cohort	Asia	Taiwan	49	23	26	Phthalates	urine, blood	T3	fMRI	13–16 years	MBP/MBzP: ↓ cingulate volume MBP/MBzP/DEHP/MEHP: ↓ cerebellum volume DEHP/MEHP/MEOHP: ↓ FA in the corpus callosum DEHP/MEHP: ↓ NQA in the corpus callosum MBzP/DEHP/MEOHP: ↓ FA in the corona radiata MBzP: ↓ NQA in the corona radiata MBP/DEHP/MEHP: ↓ FA/NQA in the SFL	NA	yes
Weng 2020	Cohort	Asia	Taiwan	59	26	33	Phthalates	urine, blood	T3	rs-fMRI/mfALFF/ReHo	13.95 ± 0.47 years	MBP: ↓ mfALFF activity in the right superior frontal gyrus and right middle frontal gyrus	MBzP: ↓ mfALFF activity in the anterior cingulum gyrus and ↓ mReHo in insula in girls	yes
Rauh 2012	Cohort	North America	USA	40	25	15	OP	cord blood, air	T3/cord blood	MRI	high exposure (8.5 ± 1.2) low exposure (7.8 ± 0.9) years	**cortical surface** ↑ superior temporal, posterior middle temporal and inferior postcentral gyri bilaterally cortical thining ↑ superior frontal gyrus, gyrus rectus, cuneus, and precuneus along the mesial wall of the right hemisphere ↓ frontal and parietal cortical thining	NA	no
van den Dries 2020	Cohort	Europe	Netherlands	518	262	256	OP	urine	T1,T2,T3	DTI	9.8 (9.6–9.9) years	DM: ↓FA & ↑ MD in white matter microstructure (most tracts except for the uncinate fasciculus tract of left hemisphere, the forceps and the corticospinal tract of the right hemisphere) DE: ↓FA (superior longitudinal fasciculus tract and corticospinal tracts) & ↑ MD (cingulate gyrus of the cingulum tract of the left hemisphere, the forceps minor tract, and the inferior longitudinal fasciculus tract of the left hemisphere) in white matter microstructure	No differences	yes
Binter 2020	Cohort	Europe	France	95	54	41	DAP, DM, DE	urine		fMRI	10.8 years	Moderate DAP: ↓ commission rate indicating improved performance DM/DE: ↓ brain activity in the left inferior and bilateral superior frontal regions during successful inhibition	No differences	
Sagiv 2019	Cohort	North America	USA	95	49	46	pesticide	pesticide exposure was estimated according 1 km radius of maternal residence during primary pregnancy (ground and air)	maternal self-report (at children 9 years) of address during pregnancy	fMRI	15–17 years	total OP pesticide use: altered brain activation during tasks of executive function total OP: ↓ bilateral brain activation in the prefrontal cortex and in the *left and right hemispheres during a cognitive flexibility task slightly ↓ bilateral activation across other regions of the frontal, temporal and parietal lobes during a cognitive flexibility task ↑ activation in the left superior parietal lobe during working memory ↑activation in the right posterior superior/middle temporal sulcus during working memory ↓ activation in the superior parietal lobule in right hemisphere during social cognition no survive FDR Survive FDR—stratified analysis by test performance (total errors and perseverative error) **Total errors: *whole results were FDR significant in high performers (compared to low performers)* ↓ Left hemisphere: superior/inferior frontal pole, broca/ba 44/45 and 44/6, dorsolateral PFC, superior/inferior temporal/postcentral gyrus, inferior parietal lobule in high performers ↓ Right hemisphere: inferior frontal pole, broca/ba 44/45, superior frontal pole/dorsolateral PFC. premotor/somatosensory cortex, posterior superior/middle temporal sulcus, inferior parietal lobule ** Perseverative errors ↓ Left hemisphere: inferior frontal pole, broca/ba 44/45 and 44/6, superior/inferior temporal/postcentral gyrus in high performers ↓ Right hemisphere: inferior frontal pole, posterior superior/middle in high performers	During language comprehension (semantic language) ↑ activation marked for the frontal and temporal regions in** males** ** left hemisphere: superior/inferior temporal/postcentral gyrus, superior parietal lobule ** right hemisphere: inferior frontal pole, broca/ba 44/45, premotor somatosensory cortex ↓ activation marked for the frontal and temporal regions in** females** ** Left hemisphere: superior parietal lobule ** Right hemisphere: inferior/superior parietal lobule no survive FDR	yes
Sagiv 2023	Cohort	North America	USA	291	158	133	DAP	urine	13- and 26-weeks’ gestation	fMRI	18.2 ± 0.3 years	↑ activation patterns in both the inferior frontal and inferior parietal lobes of the left hemisphere during a task of cognitive flexibility	No consistent	yes
Peterson 2015	Cross-sectional	North America	USA	40	23	17	PAH	air	T3	MRI	8 ± 1.3 years	↓ white matter surface in later childhood	NA	no
Liu 2023	Cohort	North America	USA	30	20	10	PAH	air	T3	MRI	7–9 years	Shyness mediated: PAH and internalizing problems ↑ shyness, predicting ↑ internalizing problems ↑ risk for internalizing problems: when children showed ↑ left ACC activity during the resolution of cognitive conflict Left ACC activity during the Simon task moderated: PAH and internalizing problems	NA	yes
Essers 2023	Cohort	Europe	Netherlands	2796	NA	NA	PAH	air	whole pregnancy	MRI	10.2 years	multipollutant analysis *APOE ε4 carriers compared to non-carriers childhood PAH: ↑ cerebral white matter volume Single-pollutant analysis—prenatal *APOEe4 carriers vs. non-carriers Cu, Fe, Si, Zn: ↓subcortical grey matter, Single-pollutant analysis—childhood *APOEe4 carriers vs. non-carriers PAH:↑ cerebral white matter volume Si:↑ cortical grey matter Cu, Fe, Si: ↓subcortical grey matter, Single-pollutant analysis—prenatal *higher vs. lower PRS for AD Cu, Fe, Si, Zn: ↓subcortical grey matter, Single-pollutant analysis—childhood *higher vs. lower PRS for AD Cu, Si: ↓subcortical grey matter, Cu: ↓corpus callosum,	NA	yes
Peterson 2022	Cohort	North America	USA	332	176	156	PAH	air	T3	MRI/DTI	6–14 years	↓ cortical thinning of dorsal parietal cortices and ↓ thickening of postero–inferior and mesial wall cortices ↓ white matter volumes, ↓ organization in white matter of the internal capsule and frontal lobe, ↑ metabolite concentrations in frontal cortex, ↓ cortical blood flow, and ↑ microstructural organization in subcortical gray matter nuclei	Associations ↑↑↑ PAH in girls	yes
Margolis 2022	Cohort	North America	Canada	40	23	17	PAH	air	T3	MRI	7–9 years	Maternal perceived stress associated with ↓ right hippocampal volume among their children Prenatal ↑ PAH moderated the association between maternal perceived stress and right CA1, CA3, and CA4/dentate gyrus volumes (↓) ↑ Right CA3 and CA4/dentate gyrus volumes: ↑ performance IQ	NA	no
Guxens 2022	Cohort	Europe	Netherlands	783 school-age children and 3,857 pre-adolescents	NA	NA	PAH	air	whole pregnancy	MRI, DTI, fMRI	6 to 10 years first wave, 9 to 12 years second wave	↓ hippocampus volume	NA	yes
Lubczyńska 2021	Cohort	Europe	Netherlands	3133	1567	1566	PAH	air (home addresses)	whole pregnancy (conception to birth)	MRI	9–12 years	↓ hippocampus volume Multipollutant analyses—subcortical structures ↓ hippocampal volume, ↓ amygdala	NA	yes
Shen 2021	Cohort	Asia	Taiwan	49	23	26	PFC (PFPS, PFOA, PFDoA, PFNA, PFUA)	urine, blood	T3	fMRI	13–16 years	PFOS/PFOA/PFNA: ↓ frontal lobe volume PFOA/PFDoA/PFNA/PFUA: ↓ cerebellum volume PFNA: ↓ GFA in the corpus callosum PFDoDA/PFNA: ↑ ISO in the corpus callosum PFOA: ↓ GFA/NQA in the internal capsule PFOA/PFDoDA: ↓ GFA in the superior longitudinal fasciculus (SFL) PFOA/PFDoDA/PFNA/PFUnDA: ↓ GFA/NQA in the external capsule	NA	yes
Weng 2020	Cohort	Asia	Taiwan	59	26	33	PFC (PFAS,PFOS)	urine, blood	T3	rs-fMRI/mfALFF/ReHo	13.95 ± 0.47 years	PFOS: ↓ mfALFF activity in the right putamen and right insula PFNA: ↓ mReHo activity in the left/right putamen and left caudate nucleus	PFOS: ↓ mfALFF activity in the right putamen and right insula in boys PFNA: ↓ mReHo activity in the left/right putamen and left caudate nucleus in boys	yes
Stewart 2003	Cohort	North America	USA	189	104	85	PCB	cord blood	birth	MRI	4.5 years	↑ percentages of errors of commission interaction: splenium size*PCB exposure ↓ splenium(children), PCB-exposed showed more than double the rate of N90commission errors compared with lesser exposed children	NA	yes
Migneron-Foisy 2022	Cohort	North America	Canada	79	45	34	PCB	blood	birth	DTI	18.4 ± 1.2 years	↑ FA of several regions of the CC, namely anterior midbody, posterior midbody, isthmus, and splenium, with the most pronounced effects observed in the splenium	NA	yes
Lamoureux-Tremblay 2021	Cohort	North America	Canada	71	40	31	PCBE	cord blood	birth	fMRI	18.31 ± 0.14 years	moderate-high cord blood PCBE vs. low exposure: ↑ conditioning phase in the right orbitofrontal cortex	NA	yes
White 2011	Cohort	Europe	Denmark	12	0	12	PCB	cord tissue	birth	fMRI	14–15 years	↑ perinatal PCBs: ↑ activation in the right posterior cingulate gyrus mixed analyses: higher perinatal PCB levels: ↑ activation in the visual cortex (than those lower levels)	Boys’ adolescents—prenatal MeHg and PCB: ↑ brain activation patterns (visual and motor tasks)	no
Margolis 2020	Cohort	North America	USA	33	18	15	PBDE	blood	T2	fMRI	5 years	Prenatal PBDEs: ↓ global efficiency (GE) of the reading-related network (RN)	NA	yes
de Water 2019	Cohort	North America	USA	47	18	29	PBDE	blood		fMRI	5.57 years	prenatal PBDE: ↑global efficiency of brain areas involved in visual attention (e.g., inferior occipital gyrus) & ↑ EF problems Higher GE of brain areas involved in visual attention: ↑ EF problems	NA	yes
Binter 2022	Cohort	North America	USA	95	49	46	DDT, DDE	blood	predictive model: prenatal	fNIRS	15–17 years	**Using predictive model—prenatal** DDE: ↑ cortical activity in the right temporal cortex during social cognition, DDE: ↑ cortical activity in the right superior parietal lobe during working memory	NA	yes
Thomason 2019	Cross-sectional	North America	USA	26	13	13	Lead	blood	birth	fMRI	23.9 to 39.6 weeks gestational age	↑ FC in cross-hemispheric, ↑ FC from posterior cingulate cortex (PCC) to lateral prefrontal cortex (PFC)	NA	no
Shen 2021	Cohort	Asia	Taiwan	49	23	26	Lead	urine, blood	T3	MRI	13–16 years	↓ frontal lobe volume / ↓ calcarine volume	NA	yes
Migneron-Foisy 2022	Cohort	North America	Canada	79	45	34	Lead	blood	birth	DTI	18.4 ± 1.2 years	There are not associated with fiber tract density No associations were found with cord and postnatal blood Pb concentrations for FA	NA	yes
Lamoureux-Tremblay 2021	Cohort	North America	Canada	71	40	31	Lead	cord blood	birth	fMRI	18.31 ± 0.14 years	No prenatal postnatal lead high vs. moderate- and low-exposed: ↑ differential activation in right dorsolateral prefrontal cortex	NA	yes
de Water 2018	Cohort	America	Mexico	15	7	8	Mn	blood	T2, T3	fMRI	6.9 ± 0.4 years	**Functional connectivity (FC)—correlation** ↓ FC between anterior cingulate cortex (ACC), orbitofrontal cortex (OFC), inferior frontal gyrus, insula and amygdala ↓ FC between ACC and these prefrontal and limbic regions ↓ FC between the insula and occipital cortex, middle temporal gyrus and angular gyrus ↓ FC between the insula and these occipito-temporal regions ↓ FC between right globus pallidus and dorsal ACC ↓ FC between right globus pallidus and dACC quadratic correlations right globus pallidus and inferior frontal gyrus	NA	yes
Migneron-Foisy 2022	Cohort	North America	Canada	79	45	34	Hg	blood		DTI	mean 18.4 ± 1.2 years	↑ FA in the posterior midbody, itsmus and splenium	NA	yes
Shen 2021	Cohort	Asia	Taiwan	49	23	26	Hg	maternal blood, cord blood	T3, birth	fMRI	13–16 years	↓ volume of the frontal and temporal lobes ↓ volume of the frontal lobes, corpus callosum and hippocampus	NA	yes
Weng 2020	Cohort	Asia	Taiwan	59	26	33	Hg	urine, blood	T3	rs-fMRI/mfALFF/ReHo	13.95 ± 0.47 years	↑activity in the left superior temporal gyrus, ↓ activity in the right caudate nucleus and right putamen in the combined group	NA	yes
Lamoureux-Tremblay 2021	Cohort	North America	Canada	71	40	31	Hg	cord blood	birth	fMRI	18.31 ± 0.14 years	high-moderate prenatal Hg vs. low-exposed: ↓ differential activation in the right and left anterior cingulate cortex during the extinction phase	NA	yes
White 2011	Cohort	Europe	Denmark	12	0	12	meHg	cord blood, maternal hair	birth	fMRI	14–15 years	mixed analyses: ↑ activation in the visual cortex (than those lower levels)	Boys’ adolescents: ↑ brain activation patterns (visual and motor tasks)	no
Shen 2021	Cohort	Asia	Taiwan	49	23	26	As	urine, blood	T3	fMRI	13–16 years	↓ frontal lobe volume / ↓ cingulate volume / ↓ cerebellum volume	NA	yes
Shen 2021	Cohort	Asia	Taiwan	49	23	26	Cd	urine, blood	T3	fMRI	13–16 years	↓ frontal lobe volume / ↓ calcarine volume	NA	yes
Guxens 2022	Cohort	Europe	Netherlands	783 school-age children and 3,857 pre-adolescents	NA	NA	Cu, Si, Zn	air	whole pregnancy	MRI, DTI, fMRI	6 to 10 years first wave, 9 to 12 years second wave	Cu, Si: ↑ global brain MD Association with Cu survived multipollutant analysis Zn: ↑ cortical surface area (precentral gyrus, precuneus, pericalcarine cortex)	NA	yes
Lubczyńska 2021	Cohort	Europe	Netherlands	3133	1567	1566	Si	air (home addresses)	whole pregnancy (conception to birth) / whole childhood (birth to MRI session 9 and 12 years)	MRI	9–12 years	Multipollutant analysis—subcortical structures ↑ hippocampal volume	NA	yes

^#^ Adjustment for socioeconomic factors (SES) were considered when the studies adjusted for education, household income or poverty

Abbreviations: MD, mean difusivity; CC, corpus callosum; IFL, inferior longitudinal fasciculus; mEP, monoethyl phthalate; mIBP, monoisobutyl phthalate; FA, fractional anisotropy; ILF, inferior longitudinal fasciculus; IFO, inferior fronto-occipital fasciculus; UF, uncinate fasciculus; HMWP, high,molecular weight phtahlates; MBP, monobutyl p[hthalate; MBzP, monobenzyl phthalate; DEHP, Di(2-ethylhexyl)phthalate; MEHP; Mono-2-ethylhexyl phthalate; SLF, superior longitudinal fasciculus; mfALFF, mean fractional amplitude of low-frequency fluctuations; ReHo, Regional Homogeneity; DM, 3-dimethyl alkyl phosphate; DE, 3-diethyl alkyl phosphate; DAP, Dialkylphosphates; ACC, anterior cingulate cortex; fNIRS, functional near-infrared spectroscopy

**Table 2:** Postnatal EDC and brain structure and/or function

Study ID	Study Design	Continent	Country	Sample size	Number Female	Number Male	Per each exposure	source of exposure	Time of exposure	Neuroimaging method	Age outcome	Findings	Sex differences	Adjustment for SES^#^
Park 2015	Cross-sectional	Asia	South Korea	115	96	19	Phthalates	urine	8 years	MRI	8.85 ± 2.32 years	DEHP: ↓ cortical thickness in the right middle and superior temporal gyri in children with ADHD	NA	no
Khodaei 2023	Cohort	North America	USA	71	39	32	OP	environment (passive dosimeter silicon wristbands)	NA	MRI, DTI	children from farmworker families (8.36 ± 0.31) and children from non-farmworker families (8.41 ± 0.32) years	NFW children had ↑ volume in several regions of white matter compared to FW children NFW children had ↑ FA in several key white matter tracts (internal and external capsule and genu) ↑ gray matter volume in selected regions of the frontal lobe medial frontal cortex, left superior frontal cortex and gyrus rectus) in NFW children than FW children white matter and gray matter findings demonstrated a ↑ degree of overlap in the medial frontal lobe, a brain region linked to decision making, error processing, and attention functions	NA	no
Bahrami 2022	Cross-sectional	North America	USA	84	40	38	Pesticide	lifetime exposure	Mean: near agricultural fields during pregnancy (0 – 9 months), early childhood (birth – 35 months), and late childhood (36 – 96 months)	fMRI	children from farmworker families 8.34 ± 0.29 and children from non-farmworker families 8.44 ± 0.35 years	Latin children: DMN → a set of regions more active during passive tasks demanding focused external attention children living in framework areas: ↑ COI (months of exposure) *DMN*clustering ↓ COI (months of exposure) *DMN*Eglob (global efficiency)	NA	yes
Mortamais 2017	Cross-sectional	Europe	Spain	242	119	123	PAH	urine	7.1–10.3y	MRI	9.7 (8.0–12.1) years	↓CN volume	NA	yes
Peterson 2015	Cross-sectional	North America	USA	40	23	17	PAH	air	5 years	MRI	8 ± 1.3 years	↑ white matter in dorsal prefrontal regions bilaterally while controlling for prenatal PAH exposure	NA	yes
Guxens 2022	Cohort	Europe	Netherlands	783 school-age children and 3,857 pre-adolescents	NA	NA	PAH	air	early and late childhood	MRI, DTI, fMRI	6 to 10 years first wave, 9 to 12 years second wave	↓ hippocampus volume	NA	yes
Migneron-Foisy 2022	Cohort	North America	Canada	79	45	34	PCB	blood	mean 11y and 18y	DTI	18.4 ± 1.2 years	↑ FA of several regions of the CC, namely anterior midbody, posterior midbody, isthmus, and splenium, with the most pronounced effects observed in the splenium FA results were mainly associated with lower RD. This study shows that exposure to Hg and PCB	NA	yes
Sussman 2022	Cohort	North America	Canada	63	23	40	POP	blood	newborn	fMRI	9–11 years	PCB: ↓ accuracy, ↓ drift rate, ↓ task-related brain activity (e.g. in inferior frontal cortex, and ↑ ADHD symptoms (e.g. hyperactivity PCB), POPs is related to task-based changes in neural activity in brain regions important for inhibitory control	NA	yes
Binter 2022	cohort	North America	USA	95	49	46	DDT, DDE	blood	childhood	fNIRS	15–17 years	DDT and DDE: altered patterns of brain activation during tasks of cognition, working memory, and executive functions DDE: ↑ cortical activation in the left inferior frontal lobe during a language comprehension and working memory tasks DDE: ↑ cortical activation in the left superior frontal lobe during a language comprehension task DDE/DDT: ↑ activation in the right frontal cortex and the right inferior parietal lobe during working memory Using predictive model—prenatal DDE: ↑ cortical activity in the right temporal cortex during social cognition, DDE: ↑ cortical activity in the right superior parietal lobe during working memory	NA	yes
Marshall 2021	Cross-sectional	North America	USA	8,524	4055	4469	Lead	Lead-risk score reflects national deciles of a weighted sum of two validated correlates of lead exposure: the ages of homes and poverty rates Lead-risk scores were previously shown to be highly associated with childhood lead exposure in children	9-10y	MRI	9–10 years	Risk of environmental lead exposure: ↓volumes of the mid-anterior, ↓mid-posterior corpus callosum ↓ volumes of these three callosal regions: poorer performance on cognitive tests (language and processing speed) Aassociation of lead exposure risk with cognitive performance was partially mediated through callosal volume, particularly the mid-posterior corpus callosum	NA	yes
Marshall 2020	Cross-sectional	North America	USA	9,712	4606	5106	Lead	blood	9-10y	MRI, fMRI	9–10 years	Children from low-income families: ↓cognitive test scores, ↓ cortical volume, ↓ cortical surface area	NA	yes
Kim 2018	Cross-sectional	Asia	South Korea	150	56	males: ADHD: 47 (62.7), healthy control: 47 (62.7)	Lead	blood	9.8y	MRI	9.8 ± 2.5 years	Significant interaction effects of DRD2 and lead exposure on the cortical thickness of the frontal lobe in ADHD ↑Left middle frontal gyrus (lateral part) ↑Superior frontal gyrus medial part)	NA	no
Cecil 2008	Cohort	North America	USA	157	74	83	Lead	blood	quarterly basis from birth until 5y, semiannually from 5 to 6.5y, again at 10y and between 15 and 17y	MRI	adult (20.8 ± 0.9), female (20.9 ± 0.9), male (20.8 ± 0.9) years	**↓** gray matter (PFC, ACC)	more pronounced for males, no significant in females	no
Brubaker 2010	Cohort	North America	USA	157	74	83	Lead	Umbilical cord blood, blood	childhood	MRI	adult (20.8 ± 0.9), female (20.9 ± 0.9), male (20.8 ± 0.9) years	**↓**gray matter volume in later ages than earlier ages of assessments. The most prominent findings was in the frontal lobes	males were more affected than females at all ages ↓↓ largest regions of gray matter were in males associated with mean blood level during 5-6y	yes
Brubaker 2009	Cohort	North America	USA	91	52	39	Lead	Umbilical cord blood, blood	childhood	DTI-MRI	adult (22.9 ± 1.5), range (20.0–26.1) years	**↓** axonal integrity and myelin organization on white matter, focally within frontal, temporal, parietal and occipital lobes **↓FA** diffuse scattered white matter regions (internal capsule, anterior and superior corona radiata) ↓MD corpus callosum & **↑**superior corona radiata Axial diffusivity:** ↓**anterior and superior corona radiata: **↓**RD corpus callosum and interna capsule & **↑** RD superior corona radiata	NA	yes
Migneron-Foisy 2022	Cohort	North America	Canada	79	45	34	Lead	blood	mean 11y and 18y	DTI	18.4 ± 1.2 years	No associations were found with cord and postnatal blood Pb concentrations for FA	NA	no
Yuan 2006	Cohort	North America	USA	42	20	22	Lead	blood	quarterly basis from birth until 5y, and again at 5.5, 6 and 6.5y	fMRI	adult 21 ± 1 years	**↓** brain activation in the left frontal gyrus and left middle temporal gyrus (regions associated with semantic language function) **↑**brain activation in the right hemisphere homolog of Wernicke’s area	NR	no
Lamoureux-Tremblay 2021	Cohort	North America	Canada	71	40	31	Lead	blood	prenatal and adolescence	fMRI	18.31 ± 0.14 years	Pb high vs. moderate- and low-exposed: ↑ differential activation in right dorsolateral prefrontal cortex (Brodmann area 9)	NA	yes
Cecil 2011	Cohort	North America	USA	159	76	83	Lead	Umbilical cord blood, blood	quarterly through 5 y of age, semiannually from 5 to 6.5 y of age, again at age 10 y and between the ages of 15 and 17 y	MRS (proton magnetic resonance spectroscopy)	adult (20.8 ± 0.9) years	**↓** gray-matter (↓NAA and Cr in basal ganglia, ↓NAA and Cho in the cerebellar hemisphere,** ↓**GLX in vermix) **↓** white-matter (**↓**Cho and GLX parietal white matter, ↓Cho in frontal white matter) - NAA, creatine and phosphocreatine Cr, Cho, mI, composite of glutamate and GLX	NA	no
Lao 2017	Cross-sectional	North America	Canada	23	low Mn exposure group: female = 8; high Mn exposure group: female = 6	low Mn exposure group: male = 5; high Mn exposure group: male = 4	Mn	tap water	childhood	MRI	12.5 ± 1.30 years	↑ volume of many areas of the basal ganglia structures (putamen, left globus pallidus) in children chronically exposed to Mn Significant correlations with fine motor performance	NA	no
Dion 2016	Cross-sectional	North America	Canada	23	14	9	Mn	tap water	childhood	fMRI	12.2 (9–15) years	↓ motor performance (high exposure group) ↓signal intensity in the globus pallidus ↓pericranial muscles pallidal index (PI) than those with lower exposure ↑ T1 relaxation time	NA	no
Baker 2015	Cohort	North America	USA	17	16	1	Mn	air	childhood	MRI	17.2 ± 4.6 years	↑ signal intensity in the basal ganglia (T1: caudate, anterior putamen, posterior putamen, and combined basal ganglia), even at very low levels of exposure	NA	no
de Water 2019	Cohort	Europe	Italy	95	49	46	Mn	dentin	early childhood	fMRI	14.63 ± 2.12 years	↑ FC between the middle frontal gyrus and medial prefrontal cortex; ↓ FC between the right putamen and pre- and postcentral gyrus ↑ FC within cognitive control brain areas, but ↓ FC between motor areas in adolescents	NA	yes
Migneron-Foisy 2022	Cohort	North America	Canada	79	45	34	Hg	blood	mean 11y and 18y	DTI	18.4 ± 1.2 years	↑ FA of several regions of the CC, namely anterior midbody, posterior midbody, isthmus, and splenium, with the most pronounced effects observed in the splenium ↑ FA splenium across the corpus callosum ↓ AD in the posterior midbody across the corpus callosum		no
Takeuchi 2022	Cross-sectional	Asia	Japan	920	359	561	Hg	hair	young adult	MRI- DTI	Male: 20.84 ± 1.92; Female: 20.54 ± 1.65 years	↓ cognitive performance (particularly on rapid processing task [speed measures]) ↓ depressive tendency ↓ regional grey matter volume (GMV) in (cluster) areas of the left thalamus and extending to left hippocampus ↓ (whole-brain) regional white matter volume (WMV) in widespread areas, including cerebellar peduncle, pontine crossing tract corticospinal tract, body and splenium of the corpus callosum, lemniscus, fornix and cingulum, internal and external capsule, anterior, posterior, and superior corona radiata, posterior thalamic radiation, sagital stratum, stria terminalis, superior longitudinal fasciculus, inferior fronto-occipital fasciculus, uncinate fasciculus, and tapetum ↑FA bilaterally distributed white matter areas overlapping with areas of significant regional WMV reduction (to mean: in a anatomically cluster spread across the left internal capsule, left posterior corona radiata, left posterior thalamic radiation, left superior longitudinal fasciculus, and left tapetum, another anatomical cluster spread across the right internal capsule, right superior corona radiata, right posterior corona radiata, right posterior thalamic radiation, right external capsule and right superior longitudinal fasciculusm and a third anatomical cluster spread across the splenium of the corpus ↓MD of the widely distributed gray and white matter areas particularly in the bilateral frontal lobe and the right basal ganglia (to mean: in an anatomical cluster that spread over a widespread area of the right lateral and medial frontal cortex, and basal ganglia, voxels spread across the right temporal pole, right amygdala, and right anterior temporal gyrus, a cluster spread across the anterior left frontal cortex, and a cluster spread across the right posterior/middle cingulum, right superior parietal lobe, and right angular gyrus).	NA	yes
Lamoureux-Tremblay 2021	Cohort	North America	Canada	71	40	31	Hg	blood	prenatal and adolescence	fMRI	18.31 ± 0.14 years	No differences between postnatal Hg exposure and brain development	NA	yes
Guxens 2022	Cohort	Europe	Netherlands	783 school-age children and 3,857 pre-adolescents	NA	NA	Zn	air	early childhood and childhood	MRI, DTI, fMRI	6 to 10 years first wave, 9 to 12 years second wave	↑ cortical surface area (right precentral gyrus, precuneus, and pericalcarine cortex) and increased the volume of nucleus accumbens	NA	yes
Pujol 2016	Cohort	Europe	Spain	263	127	136	Cu	air	childhood	DTI, fMRI	9.7 ± 0.9 years	↓ motor performance and altered structure of the basal ganglia (specifically, caudate nucleus region in terms tissue composition and neural track water diffusion) ↑Gray matter concentration L caudate nucleus; ↑Fractional anisotropy DTI L caudate nucleus; ↓Functional connectivity L frontal cortex to L caudate ↓ reciprocal connectivity between the caudate nucleus and the frontal cortex	NA	no

^#^ Adjustment for socioeconomic factors (SES) were considered when the studies adjusted for education, household income or poverty

Abbreviations: MD, mean difusivity; CC, corpus callosum; IFL, inferior longitudinal fasciculus; mEP, monoethyl phthalate; mIBP, monoisobutyl phthalate; FA, fractional anisotropy; DEHP, Di(2-ethylhexyl)phthalate; MEHP; Mono-2-ethylhexyl phthalate;; fNIRS, functional near-infrared spectroscopy; NAA, N-acetyl aspartate; GLX,; Cho, glycerolphosphocholine and phosphocholines; mI, myo-inositol; GLX, glutamine; DMN, Default mode network; MD, Mean diffusivity; RD, Radial diffusivity; CN. Caudate nucleus

### Prenatal Exposure to EDCs (Table 1)

#### Phenols

Two studies examined the impact of prenatal BPA exposure (measured in maternal urine) on child brain development [18, 20]. Using structural MRI (*n* = 87), Zheng et al. found that higher prenatal BPA exposure was associated with decreased brain volumes in the opercular region of the inferior frontal gyrus, the superior occipital gyrus, and the postcentral gyrus in children at 4 years [20]. The limbic system showed alterations, with increased volume in the hippocampus, entorhinal area, and amygdala, but decreased volume in the accumbens area [20]. There were hemispheric differences in associations with BPA exposure [20]. Using DTI (*n* = 98), Grohs et al. reported that higher prenatal exposure to BPA was associated with changes in brain microstructure, notably reflected in increased mean diffusivity (MD) in regions like the splenium and the right inferior longitudinal fasciculus (ILF) in children at 4 years [18]. Increased MD in the splenium mediated the relationship between maternal prenatal levels of BPA and children's internalizing behavior [18].

These findings suggest that prenatal exposure to BPA may influence limbic system; however, these studies had relatively small sample size and were limited to BPA only (and not newer replacement of concern [21]).

#### Phthalates

Four studies examined prenatal phthalates exposure (measured in maternal urine) [21–24]. Ghassabian et al. (*n* = 775) showed that higher prenatal phthalate exposure (e.g., monoethyl phthalate (mEP)) was associated with decreased total gray matter volume at 10 years [22]. Total grey matter volume partially mediated the relationship between maternal prenatal mEP and children’s cognition [22]. England-Mason et al. used DTI (*n* = 76) and found that prenatal phthalates exposure was associated with decreased FA in the left ILF and increased MD in the right inferior fronto-occipital fasciculus (IFO), right pyramidal fibers, left and right uncinate fasciculus at 4 years [21]. MD in the right IFO indirectly mediated the relationship of prenatal high molecular weight phthalate exposure with internalizing and externalizing problems [21]. Using fMRI, Shen et al. (*n* = 49) reported that prenatal phthalate exposure was associated with decreased cingulate and cerebellum volume at 13 years [23]. Higher phthalate exposure was also associated with decreased FA and quantitative anisotropy (QA) in the corpus callosum (CC), the corona radiata, and the superior longitudinal fasciculus (SLF) [23]. Weng et al. (*n* = 59) found that maternal urinary monobutyl phthalate (MBP) levels during pregnancy was associated with reduced mean fractional amplitude of low-frequency fluctuations (mfALFF) in the right superior frontal gyrus and right middle frontal gyrus in teenagers [24].

##### Sex Differences

Ghassabian et al. (*n* = 775) reported that higher prenatal monoisobutyl phthalate (mIBP) concentrations were associated with decreased cerebral white matter volume among girls only, and white matter volume mediated the relationship between prenatal mIBP exposure and lower cognition [22]. Weng et al. (*n* = 59) found that higher prenatal monobenzyl phthalate (MBzP) was associated with reduced mfALFF in the right putamen and reduced mean regional homogeneity (mReHo) in the insula only in girls [24].

Taken together, prenatal exposure to phthalates may influence brain structures and neural pathways associated with emotional, behavior and cognitive development. Moreover, sex differences were reported in these studies.

#### OPs

We found five studies on prenatal OPs exposure and child brain development. These studies measured OPs in blood [25, 26], urine samples [27, 28], or defined risk of OP exposure based on the proximity of pregnancy residential addresses to agricultural communities [29]. In a prospective study using structural MRI, Rauh et al. (*n* = 40) found that elevated cord blood chlorpyrifos concentrations were associated with increased superior temporal, posterior middle temporal and inferior postcentral gyri bilaterally cortical thinning, and decreased frontal and parietal cortical thinning at 8 years [26]. Using DTI, van den Dries et al. (*n* = 518) found that prenatal exposure to OPs (as measured by urinary metabolites 3-dimethyl(DM) and 3-diethyl(DE) alkyl phosphate) were associated with decreased FA in SLF tracts and corticospinal tracts at 9 years [28]. They also reported increased MD in cingulate gyrus of the cingulum tract, and the ILF tract of the left hemisphere [28]. Binter et al. (*n* = 95) used fMRI and reported that maternal urinary concentration of dialkylphosphates (DAP) during pregnancy was associated with reduced commission rate, suggesting enhanced performance at 11 years. Prenatal DM and DE levels in urine were associated with decreased activity in the left inferior and bilateral superior frontal regions during inhibition processing [27]. Using fMRI, Sagiv et al. (*n* = 95) reported that children high risk of exposure to pesticides had altered brain activation during tasks of executive function at 16 years [29]. During cognitive flexibility tasks, high risk adolescents showed decreased bilateral activation within the PFC and across both hemispheres; and decreased bilateral activation across frontal, temporal, and parietal lobes [29]. Using fMRI in another study, Sagiv et al. (*n* = 291) found that maternal urinary DAP levels during pregnancy were associated with increased activation patterns in both the inferior frontal and inferior parietal lobes of the left hemisphere during cognitive flexibility tasks at 18 years [25].

##### Sex Differences

Sagiv et al. (*n* = 95) found that boys with high risk of exposure to pesticides had higher activation in the frontal and temporal regions during the semantic language tasks; while this relationship was inverse among girls [29].

Overall, OP pesticides examined in these studies were heterogeneous. However, these studies cautiously suggest that prenatal exposure to OPs are associated with changes in executive functioning during childhood and adolescence.

#### PAHs

We identified seven studies on prenatal PAH exposure and neuroimaging. These studies estimated PAH exposure with air sampling or relied on geospatially derived estimates [30–36]. In a study of 40 mother-child pairs, Petterson et al. found that children with high PAH exposure during fetal period had decreased white matter surface at 8 years [30]. In another study, Liu et al. (*n* = 30) reported that children with high prenatal exposure showed increased left anterior cingulate cortex (ACC) activity during the resolution of cognitive conflict [31]. Esser et al. (*n* = 2796) did not find association between higher prenatal PAH levels and brain measures in pre-adolescence, but there was a moderation by genetic risk for Alzheimer’s Disease [32]. Using structural MRI and DTI, Peterson et al. (*n* = 332) found that higher prenatal PAH levels were associated with decreased cortical thinning of dorsal parietal cortices, postero-inferior and mesial wall cortices at 8 years. Children exposed to higher PAH levels during pregnancy had smaller white matter volumes, reduced organization in white matter of the internal capsule and frontal lobe, higher metabolite concentrations in frontal cortex, reduced cortical blood flow, and greater microstructural organization in subcortical gray matter nuclei [33]. Margolis et al. (*n* = 40) found that prenatal PAH levels moderated the association between maternal perceived stress and right CA1, CA3, CA4 and dentate gyrus volume at 8 years [34]. Guxens et al. (*n* = 783) and Lubczynska et al. (*n* = 3133) reported that higher prenatal PAH exposure was associated with decreased hippocampus volume [35, 36].

##### Sex Differences

In one study, the relationship between prenatal PAH levels and brain structure were stronger among girls than boys [33].

In sum, these studies suggest that prenatal PAH levels is negatively associated with brain structure, mainly white matter volume, subcortical grey matter, hippocampus and amygdala volume in childhood and adolescence.

### Persistent Organic Pollutants (POPs)

Two studies investigated prenatal PFAS exposure relying on concentrations in maternal serum [23, 24]. Shen et al. (*n* = 49) found that prenatal PFAS exposure was associated with decreased frontal lobe and cerebellar volume in adolescents [23]. Higher maternal PFAs levels were associated with decreased FA in the CC, the SLF, the internal and external capsule, and decrease QA in the internal and external capsule [23]. Weng et al. (*n* = 59) found that maternal serum perfluorooctane sulfonate (PFOS) levels during pregnancy were associated with decreased mfALFF activity in the right putamen and right insula in adolescents [24]. Higher PFAS levels was associated with decreased mReHo activity in the putamen and left caudate nucleus in adolescents [24].

#### Sex Differences

The inverse association between PFOS exposure and mfALFF activity in the right putamen and right insula was more pronounced among boys. Similar sex differences were observed for mReHo activity in the putamen and left caudate nucleus [24].

Five studies focused on PCB levels in maternal and cord blood samples [37–40] and cord tissue [41]. Stewart et al. (*n* = 189) found that higher perinatal PCBs levels were associated smaller splenium at 4 years [39]. Using DTI, Migneron-Foisy et al. (*n* = 79) found that perinatal PCBs levels are associated with increased FA in CC, with the most pronounced effect was in the splenium [38]. Lamoureux-Tremblay et al. (*n* = 71) used fMRI and reported that higher perinatal PCB levels were associated with increased conditioning phase in the right OFC [37]. Using fMRI, Sussman et al. (*n* = 63) found that early pregnancy PCB levels were associated with decreased accuracy, drift rate and task-related brain activity in the inferior frontal cortex at 10 years [40]. These regions were associated with task-based changes in neural activity within brain regions crucial for inhibitory control [40].

#### Sex Differences

White et al. (*n* = 12) reported that higher perinatal PCB levels were associated with increased activation in the right posterior cingulate gyrus among adolescents boys only [41].

One study examined OC levels in maternal blood and fMRI measures in childhood [42]. Binter et al. (*n* = 95) reported that higher prenatal dichlorodiphenyldichloroethylene (DDE) levels were associated with increased cortical activity in the right temporal cortex during social cognition and in the right superior parietal lobe during a working memory task [42].

In summary, prenatal POPs levels was negatively associated with brain structure, including basal ganglia structures in childhood and adolescence.

### Heavy Metals

#### Lead

Four studies examined prenatal lead exposure (measured in maternal and cord blood) [23, 37, 38, 43]. Thomason et al. examined the relationship between maternal lead levels and fetal brain fMRI measures (*n* = 26). They reported increased functional connectivity (FC) in cross-hemispheric communication and from the posterior cingulate cortex (PCC) to the lateral prefrontal cortex (PFC) in fetal brain in relation to higher lead exposure [43]. Shen et al. (*n* = 49) found that higher prenatal lead levels are associated with decreased frontal lobe and calcarine volume in adolescence [23]. Using DTI and fMRI, Migneron-Foisy et al. (*n* = 79) and Lamoureux-Tremblay et al. (*n* = 71) did not find associations between perinatal lead exposure and neuroimaging findings at 18 years [37, 38].

#### Manganese (Mn)

We found one study that examined prenatal Mn exposure in relation to child brain development. de Water et al. (*n* = 15) used measures of Mn in blood and dentin samples [44]. They reported associations between higher prenatal Mn exposure and decreased FC in critical brain regions involved in emotional regulation and cognitive tasks in fMRI of children aged seven. Specifically, decreased FC was observed between the ACC, orbitofrontal cortex (OFC), inferior frontal gyrus, and amygdala, suggesting broader impacts on emotion regulation networks. Additionally, they found reduced FC between the insula and occipital cortex, middle temporal gyrus and angular gyrus, alongside shifts in FC with occipito-temporal regions with higher Mn exposure [44].

#### Mercury (Hg)

Five studies examined prenatal Hg exposure and neuroimaging. These studies measured Hg in maternal hair, (cord) blood or urine samples [23, 24, 37, 38, 41]. Migneron-Foisy et al. (*n* = 79) found that elevated cord blood Hg concentrations were associated with increased FA in the posterior midbody, itsmus and splenium across the CC at 18 years [38]. Shen et al. (*n* = 49) reported that higher perinatal Hg exposure was associated with smaller volume in the frontal and temporal lobes, the CC and right hippocampus at age 13 [23]. Weng et al. (*n* = 59) found that higher prenatal exposure to MeHg was associated with increased activity in certain brain areas at age 13, such as the superior temporal gyrus, caudate nucleus and putamen [24]. Lamoureux et al. (*n* = 71) reported that high-moderate prenatal exposure to Hg was associated with lower differential activation in the right and left anterior cingulate during the extinction phase at 18 years [37].

##### Sex Differences

White et al. (*n* = 12) reported that perinatal MeHg exposure was associated with increased brain activation patterns during visual and motors tasks among adolescents boy only [41]. Additionally, a mixed analyses revealed that children with higher perinatal MeHg and PCB levels had increased brain activation in the visual cortex than those with lower levels among boys [41].

#### Other Heavy Metals

Shen et al. (*n* = 49) examined the relationship of arsenic (As) and cadmium (Cd) levels in cord blood samples and adolescents’ brain structural measures [23]. Perinatal As levels was associated with decreased frontal lobe, cingulate and cerebellar volume in adolescents. Perinatal Cd exposure was associated with decreased frontal lobe and calcarine volume in adolescents [23].

Guxens et al. (*n* = 783) estimated prenatal exposure to heavy metals, such as copper (Cu), silicon (Si), and Zinc (Zn), using geospatial-based air pollution models [36]. They found that higher prenatal Cu and Si levels were associated with increased global brain MD in pre-adolescents. The association with Si but not Cu remained in multipollutant analysis. Higher prenatal Zn levels were associated with increased cortical surface area (precentral gyrus, precuneus, and pericalcarine cortex) in pre-adolescents [36]

Similarly***,*** Lubczyrisska et al. (*n* = 3133) estimated metals in air quality and performed a multipollutant analysis. They found that higher prenatal Si levels were associated with increased amygdala and hippocampal volumes in adolescents [35].

These studies highlight the impact of prenatal Hg exposure on brain structure and function throughout adolescence and into young adulthood. However, neuroimaging studies of perinatal exposure to other heavy metals remain limited.

In sum, prenatal exposure to EDCs (such as phthalates, OPs, PAH and Hg) may lead changes in brain structure and function. Mainly, those neural pathways associated with emotional regulation, cognitive development and executive functioning in childhood and adolescence.

### Childhood Exposure to EDCs (Table 2)

#### Phthalates

A cross-sectional study examined the association of urinary phthalates metabolites and child brain measures [45]. Using structural MRI (*n* = 115), Park et al. found that in 8-year-old children with attention-deficit/hyperactivity disorder (ADHD), higher urinary levels of DEHP metabolites were associated with thinner cortices in the right middle and superior temporal gyri [45].

#### OPs

Two neuroimaging studies examined postnatal OPs exposure. In both studies, the risk of exposure was defined based on the presence of at least one adult in the household who had been employed at a non-organic farm [46, 47].

Using structural MRI and DTI, Khodaei et al. (*n* = 71) reported that children living in non-farmworker families had increased volume in gray matter structures (frontal lobe medial frontal cortex, left superior frontal cortex and gyrus rectus) and several white matter structures than those living in farmworker families [46]. Moreover, children with high risk of exposure had increased FA within internal and external capsule compared to low risk. Notably, the white matter and gray matter findings demonstrated an increased degree of overlap in the medial frontal lobe, a brain region predominantly linked to decision making, error processing, and attention functions [46]. Using fMRI, Bahrami et. al (*n* = 84) found that 8-year old children living for longer period in farm communities had decreased default mode network activity during passive task requiring external attention, as well as reduced global efficiency [47].

These studies suggest the potential impact of postnatal OP exposure on brain structure and function during executive function and attention processes throughout childhood period. Further research is needed with large sample size and relying on better characterization of exposure with repeated measures.

#### PAHs

Three studies examined postnatal PAHs exposure, one measured PAHs concentrations in urine sample [36, 48] and the other estimated exposure in air quality [30, 36]. In two cross-sectional study using structural MRI, Mortamais et al. (n=242) and Perterson et al. (n=40) found that children with higher PAH levels had decreased caudate nucleus volume at 10 years [48] and increased white matter in dorsal prefrontal regions [30]. In a longitudinal study (n=783), Guxens et al. did not find any association between childhood PAH levels and brain structure in pre-adolescents [36]. These inconsistent findings suggest that more studies are needed to unravel the impact of childhood PAH levels on brain development.

#### POPs

One longitudinal studies examined postnatal PCB exposure, using blood levels [38]. By using DTI, Migneron-Foisy et al. (*n* = 79) found that higher childhood PCBs were associated with increased FA of several regions of the CC at 18 years [38].

One longitudinal study examined the impact of postnatal OC exposure (measured in blood) on brain development [42]. Using fMRI, Binter et al. (*n* = 95) found higher childhood DDE and dichlorodiphenyltrichloroethane (DDT) levels were associated with increased cortical activation in the frontal lobe during language comprehension and working memory tasks in adolescents [42].

These findings underscore the impact of postnatal lead exposure on both gray and white matter integrity, suggesting potential implications for neurodevelopmental processes and cognitive function.

### Heavy Metals

#### Lead

We identified ten studies on postnatal lead exposure and brain. These studies measured lead in the blood samples [37, 38, 49–54] or estimated lead-risk score based on the age of homes and poverty rates [55, 56].

Marshall et al. (*n* = 8524) reported that nine-year-old children with high lead-risk score had reduced volumes in mid-anterior and mid-posterior CC compared to low risk groups [56]. CC volume mediated the association between lead-risk exposure and poor cognitive performance. In another publication (*n* = 9712), Marshall et al. reported that children from low-income families with high lead-risk score had lower cognitive scores, alongside diminished cortical volume and cortical surface area [55]. In 150 children at 10 years, Kim et al. found that lead exposure interacts with genetic factors, such as the dopamine receptor D2 gene, impacting cortical thickness in the frontal lobe among children with ADHD [54]. In longitudinal studies using structural MRI, Cecil et al. (*n* = 157) and Brubaker et al. (*n* = 157) found that childhood lead exposure was associated with decreased gray matter volume, particularly in crucial cognitive processing areas, such as the PFC and ACC at 21 years [50, 52]. Brubaker et al. (*n* = 91) used DTI and founded increased childhood blood lead levels were associated with decreased FA across various brain regions at 22 years [51]. Additionally, in regions of the corona radiata, exposure was associated with decreased FA and axial diffusivity (AD) and increased MD and radial diffusivity (RD) [51]. However, Migneron-Foisy et al. (*n* = 79) did not find any association between postnatal lead exposure and FA at 18 years [38]. In fMRI studies, Yuan et al. (*n* = 42) found that higher childhood exposure to lead was associated with reduced activation in areas associated with language function, alongside compensatory increases in other regions at 21 years [49]. Lamoureux-Tremblay et al. (*n* = 71) reported that adolescent exposed to lead had increased differential activation in right dorsolateral PFC at 18 years [37]. Cecil et al. (*n* = 159) showed higher childhood lead exposure was associated with significant alterations of metabolites levels in both gray and white matter regions at 21 years [53].

##### Sex Differences

According to two longitudinal studies, males exposed to lead throughout childhood were more affected than female [50, 52].

These findings underscore the impact of postnatal lead exposure on both gray and white matter integrity, suggesting potential implications for neurodevelopmental processes and cognitive function.

#### Mn

We identified four studies on postnatal exposure to Mn (based on air monitoring, or measures in tap water and dentin samples) [57–60].

In a cross-sectional study that used structural MRI, Lao et al. (*n* = 23) reported that children exposed to Mn through drinking water over extended periods had alterations in basal ganglia structures at age 13, including the enlargement of the putamen and left globus pallidus [59]. In another cross-sectional study, Dion et al. (*n* = 23) found that children highly exposed to Mn showed lower signal intensity for the pallidal index calculated with pericranial muscles in fMRI at aged 12 [58]. Both studies reported that motor performance was low in high-exposed groups [58, 59].

Baker et al. (*n* = 17) found that children exposed to Mn, even at very low exposure, had a higher signal intensity in the basal ganglia structures at 17 years [57]. Using fMRI, Water et al., (*n* = 95) reported that postnatal Mn exposure was associated with increased FC between middle frontal gyrus and medial PFC [60].

While findings suggest postnatal exposure to Mn may be associated with alterations in the basal ganglia structures, implicated in low (fine) motor performance, these findings should be interpreted cautiously since these studies had small sample sizes.

#### Hg

We found four studies that examined the impact of postnatal Hg exposure and brain development. These studies measured Hg in blood and hair samples [38, 41, 61].

In a longitudinal study using DTI, Migneron-Foisy et al. (*n* = 79) reported that higher postnatal (mean of child and adolescence) blood Hg levels were associated with increased FA splenium and decreased AD in the posterior midbody across the CC at 18 years [38]. In a cross-sectional study, Takeuchi et al. (*n* = 920) found that Hg exposure was associated with decreased regional white matter volume at 21 years, such as major white matter tracts (CC, internal and external capsule, corona radiata, posterior thalamic radiation, sagital stratum and fasciculus) and specific white matter pathways (cerebellar peduncle, lemniscus, fornix and cingulum) [61]. Higher Hg exposure was associated with increased FA in white matter regions bilaterally, overlapping with areas exhibiting reductions in regional white matter volume. This study reported associations with decreased MD of gray and white matter areas specifically in the bilateral frontal lobe and the right basal ganglia [61]. In a longitudinal study, Lamoureux et al. (*n* = 71) did not find association between adolescence exposure to Hg with fMRI measures at 18 years [37].

These previous studies highlight the influence of postnatal Hg exposure on brain structure and function throughput adolescence and into young adulthood.

#### Other Heavy Metals

Guxens et al. (*n* = 783) estimated childhood exposure to Zn via air pollution [36]. They found that higher childhood Zn exposure was associated with increased cortical surface area (right precentral gyrus, precuneus, and pericalcarine cortex) and increased the volume of nucleus accumbens in pre-adolescents [36]. Pujol et. al. found higher Cu levels was associated with decreased motor performance and altered structures of basal ganglia at 9 years [62].

## Discussion

**Fig. 1:**
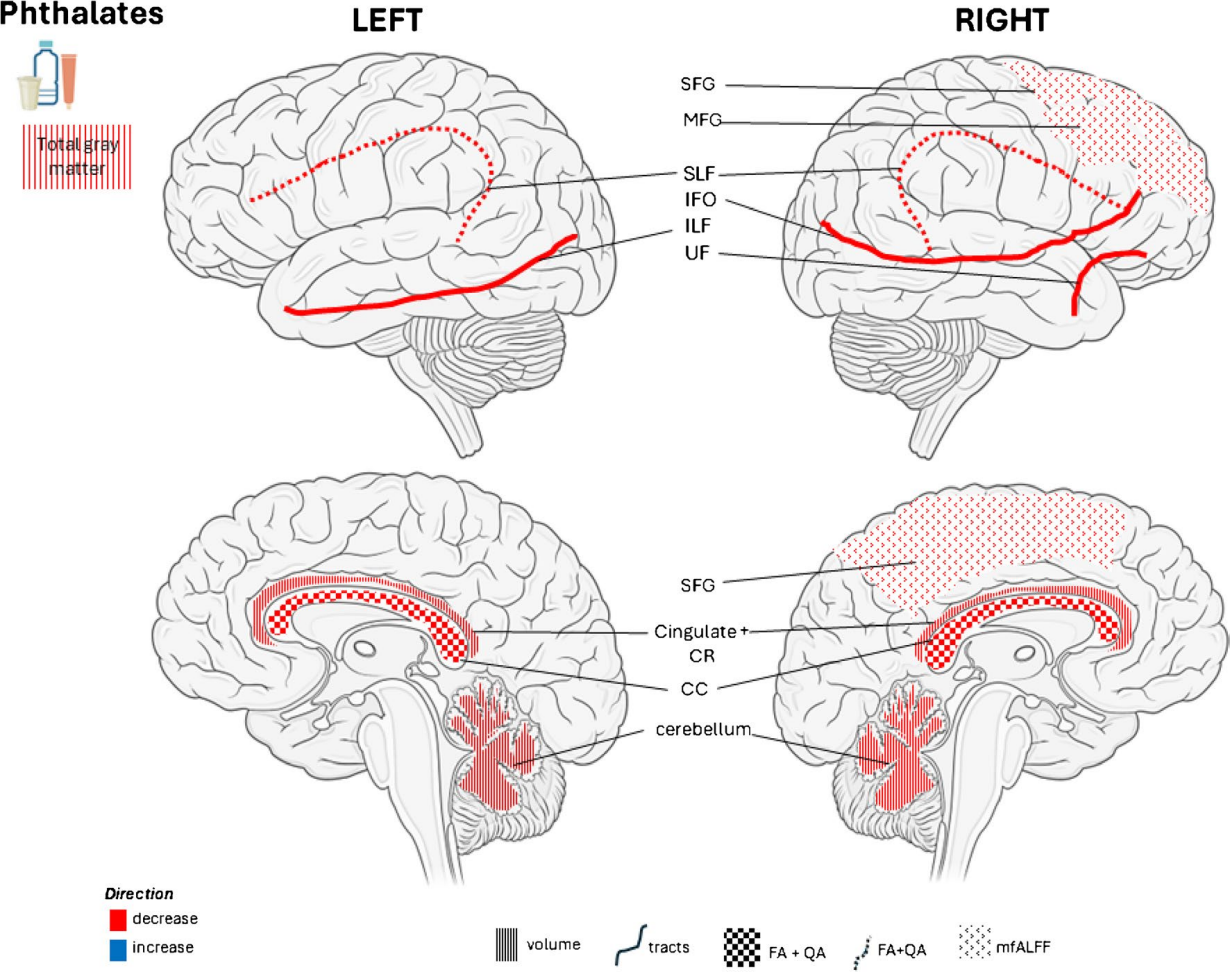
Prenatal Phthalates exposure and Brain morphology. Abbreviations: ILF, Inferior longitudinal fasciculus; IFO, inferior fronto-occipital fasciculus; UF, uncinate fasciculus; FA, fractional anisotropy; QA, quantitative anisotropy; SLF, Superior longitudinal fasciculus; CR, corona radiata; SFG, superior frontal gyrus; MFG, middle frontal gyrus; mfALFF, mean fractional amplitude of low-frequency fluctuations

**Fig. 2:**
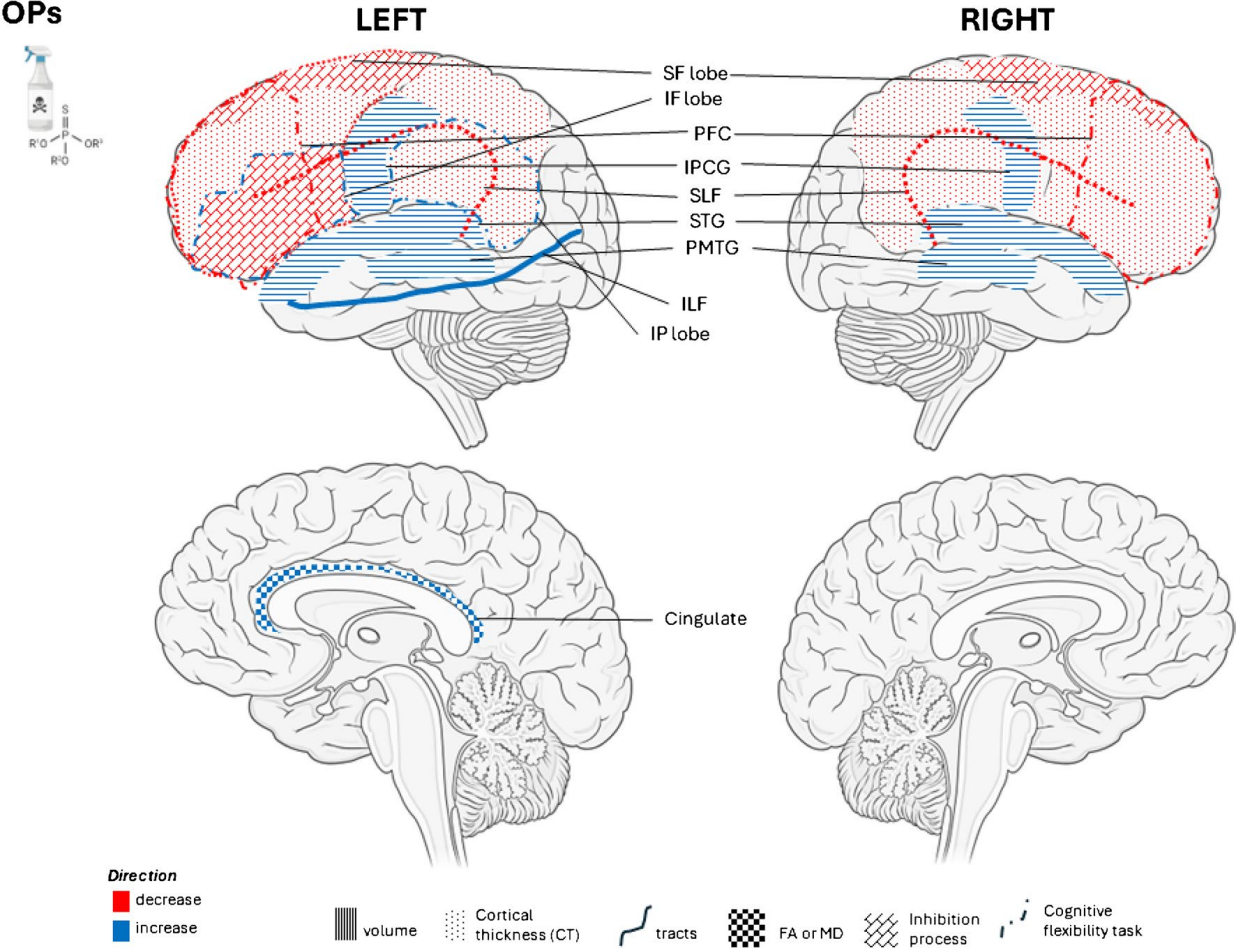
Prenatal OP exposure and Brain morphology. Abbreviations: OP, organophosphates; SF lobe, superior frontal lobe; IF lobe, inferior frontal lobe; PFC, prefrontal cortex; IPCG, inferior postcentral gyrus; SLF, superior longitudinal fasciculus; STG, superior temporal gyrus; PMTG, posterior middle temporal gyrus; ILF, Inferior longitudinal fasciculus; IP lobe, inferior parietal lobe; FA, fractional anisotropy; MD, mean diffusivity

**Fig. 3:**
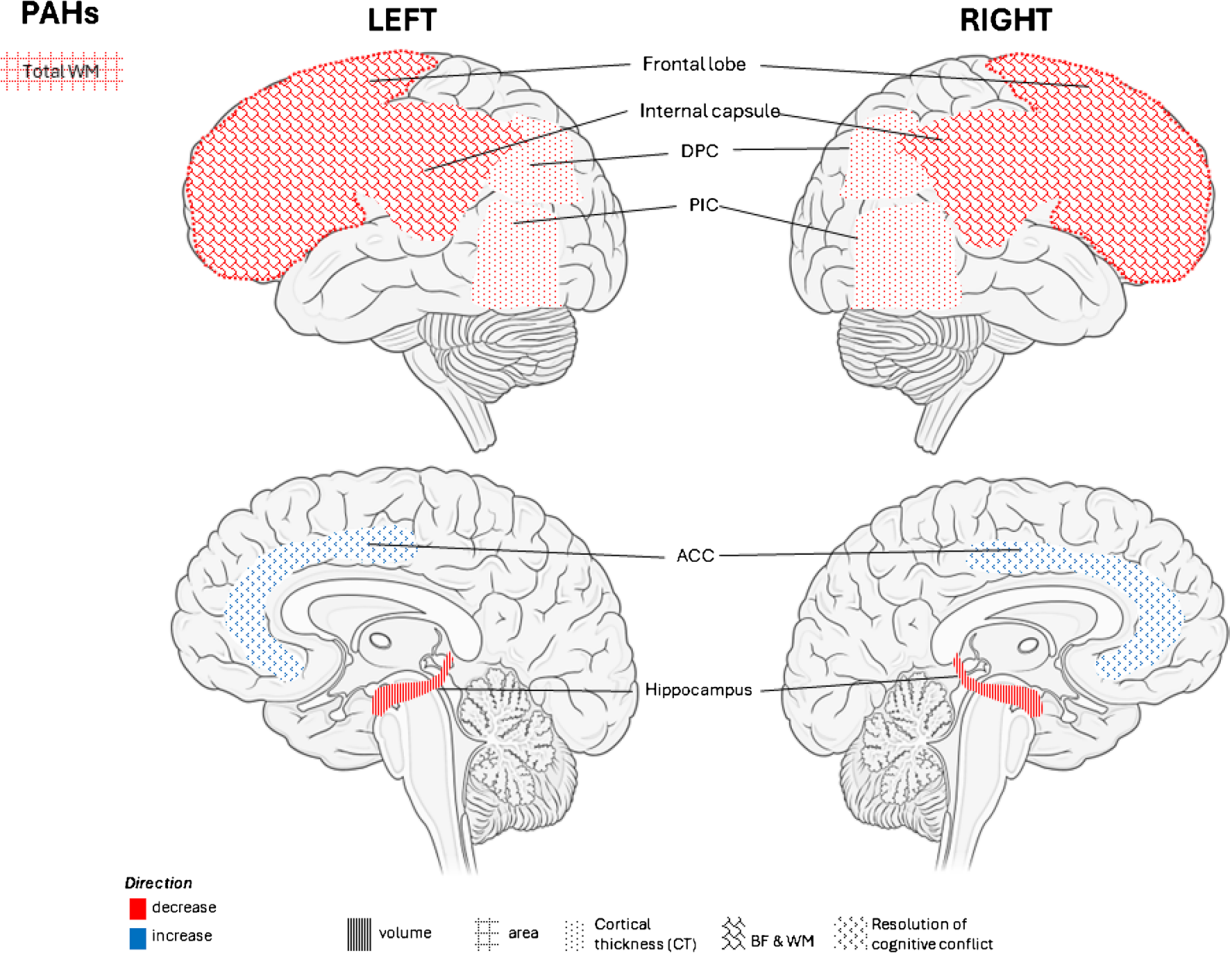
Prenatal PAH exposure and Brain morphology. Abbreviations: PAHs, polycyclic aromatic hydrocarbons; DPC, dorso-parietal cortex; PIC, posterior-inferior cortex; ACC, anterior cingulate cortex; BF, blood flow; WM, white matter

Among 46 articles reviewed here, we found several studies with a longitudinal design that show associations between early life exposure to EDCs and brain structural or function as assessed by MRI. Critical periods were characterized by adaptive, dynamic, and sensitive brain development, including pregnancy, childhood, and adolescence. Overall, prenatal exposures to EDCs, i.e., phthalates, OPs, PAHs and POPs, were associated with altered brain development, including alterations in total white and gray matter volume, cortical thickness, basal ganglia, hippocampus and amygdala in childhood and pre-adolescence See illustration of brain influences of prenatal EDC exposure (see Figs. 1, 2 and 3). Research studies focusing on prenatal exposure to phthalates, OPs or PAHs had yielded more conclusive findings compared to studies examining other EDCs [21, 22, 28, 30, 36]. Additionally, prenatal exposure to EDCs (in particular OPs) was associated with reduced neural activity in regions involved executive function and inhibitory control by using fMRI [25, 27, 29]. However, findings on postnatal EDCs exposure were inconsistent. A few studies performed mediation analysis to examine how total white and grey matter volume mediated the association between prenatal exposure to EDCs (i.e., phthalates) and cognitive and behavior problems [21, 22]. Larger epidemiological studies that used geospatial models to estimate PAH exposure showed associations with decreased white matter and structures of limbic system [33, 35, 36]. Additionally, evidence presented here supports the notion that the brain influences of EDCs show sexual dimorphic patterns. For example, only girls exposed to phthalates prenatally had decreased white matter volume [22], whereas boys exposed to prenatal PAHs shown lesser effects on white matter volume than girls [33], and boys exposed to prenatal OPs levels had higher activation of in the frontal and temporal regions during semantic tasks [29]..

Since brain development begins during pregnancy and follows up in the postnatal period, including infancy and adolescence, more studies are needed to explore how early exposures continue to shape neural pathways and brain plasticity, underlying nature and nurture for brain development. Although some investigations examined the relationship between prenatal exposure to EDCs and brain development, suggesting that prenatal exposure to EDCs (primarily, phthalates, PAHs, OPs, and Hg) may influence brain development later in life, there is much limited literature on postnatal exposure to EDCs and brain development. Few existing studies on exposure during childhood and adolescence focused on exposure to lead only, highlighting the need for more studies.

Exposure to EDCs during pregnancy may influence on brain development in womb and exposure, which impacts brain development in utero and primarily sets the stage for future growth. Early life exposures continue to shape neural pathways and brain plasticity, underscoring the importance of a nurturing and healthy environment for optimal brain development.

### Methodological Strengths and Opportunities

Among 46 MRI studies that examined brain influences of EDCs, 65% were prospective, allowing a temporal relationship between exposure and outcome. Most studies measured EDCs (or their metabolites) in biological samples (70%), and applied a multipollutant approach (10%) which added to the robustness of their findings [63]. Despite these methodological strengths, these investigations had limitations that should be addressed in future studies. First, several studies had small **sample size** (less than 50 participants), which might reduce the statistical power in the results and increase the risk of type II error [64, 65]. Small sample size could increase the possibility of outliers driving the associations that could not be replicated in other samples [66]. This is particularly an issue for EDCs that might influence brain development through sexual dysmorphic mechanisms since small studies would not allow for testing for interaction by sex [67]. Second, **timing and frequency of exposure assessments** should be discussed. Measurement of exposure (whether through measurements of EDCs or their metabolites in biospecimens, or in the air) in different windows of brain development allows identification of periods of susceptibility [9, 10, 68]. Additionally, incorporating repeated measures of exposure would be essential to reduce the measurement error, particularly for EDCs that are non-persistent and have short half-life (e.g., phthalates) [22, 64]. With regards to windows of brain development, prenatal exposure during the third trimester is important because of significant changes in brain morphology happen during this period (critical period) [10]. Whereas (epi)genetic influences may occur during the periconceptional period (sensitive period), and subsequently alter brain development [69, 70]. Third, the challenge to summarize research findings is amplified by the heterogeneity in the **outcome assessment** across studies. Differences in brain MRI modalities contribute to this complexity, resulting in inconsistent results when attempting to combine different modalities, brain structures/regions and age of the assessment. More studies are needed to consider these methodological issues.

MRI scans reveal that these early exposures can alter brain architecture, affecting areas responsible for cognitive functions, emotional regulation, and behavioural responses. However, most of these studies involved relatively small populations and lacked diversity in population characteristics. This underscores the need for careful interpretation of findings and consideration of the strengths and limitations inherent in each research approach.

Further research studies are needed to enhance our comprehension and increase the generalizability of these findings. We could improve our comprehension of the underlying mechanism and nuance involved in devolving more diverse populations and employing standardized methodologies. First, to direct research questions and ensure methodological rigor during the research, it is crucial to formulate hypotheses before conducting studies. Clear hypothesis formulation helps establish a focused framework for research and aids the interpretation of study outcomes. Additionally, it is essential to prioritize longitudinal studies with diverse populations that rigorously follows methodological considerations, such as adjustment for confounding variables and correction for multiple comparisons. The use of multipollutant analysis may yield deeper insights regarding the impact of combined or cumulative effect of EDCs on brain development. Finally, researchers should strive for greater homogeneity in the methodology including exposure and outcome assessment regarding the relationship of EDCs and brain imaging to improve the comparability and reliability of studies.

## Conclusions

These findings underscore the profound and lasting effects of prenatal EDC exposure on brain development, emphasizing the need for better regulation and strategies to reduce exposure and mitigate impacts.

### Supplementary Information

Below is the link to the electronic supplementary material.

ESM 1 (DOCX 115 KB)

## Data Availability

No datasets were generated or analysed during the current study.
