# Supplementary material for: Exposure to Endocrine Disruptors in Early life and Neuroimaging Findings in Childhood and Adolescence: a Scoping Review
Source: Curr Environ Health Rep. 2024 Jul 30;11(3):416–42. doi: 10.1007/s40572-024-00457-4 (PMC11324673; doi:10.1007/s40572-024-00457-4)

**Supplementary material**

Exposure to Endocrine Disruptors in Early life and Neuroimaging Findings in Childhood and Adolescence: A Scoping Review

*Running title: Early life endocrine disruptors and neuroimaging*

Kim N Cajachagua-Torres^1,2^, Hugo G Quezada-Pinedo^2^, Tong Wu^3^, Leonardo Trasande^1,4^,

Akhgar Ghassabian^1,4^

^1^ Department of Pediatrics, NYU Grossman School of Medicine, New York, NY, the United States.

^2^ Department of Pediatrics, Erasmus MC, Erasmus University Rotterdam, Rotterdam, the Netherlands.

^3^ Department of Radiology and Nuclear Medicine, Erasmus MC, Erasmus University Rotterdam, Rotterdam, the Netherlands.

^4^ Department of Population Health, NYU Grossman School of Medicine, New York, NY, United States.

**Contents**

1. **eMethods. Literature search strategy using the PubMed and PsycINFO**
2. **eMethods. Supplemental methods**
3. **sFigure 1. Flowchart of literature review**
4. **eMethods.** **Literature search strategy using the PubMed and PsycINFO**

(((embryo or embryus or fetus or fetal or foetus or foetal or prenatal or pregnancy or infant or preschool or toddler or child* or puber* or adolescen* or teenager) AND exposure) or prenatal exposure delayed effects) AND (Plastic* or Endocrine disruptors or EDCs or Phenols or Bisphenol or Bisphenyl or Phthalates or Perchlorate or Pesticides or organophosphates or atrazine or DDT or 2,4-D or glyphosate or PFAS or Perfluoroalkyl or polyfluoroalkyl or Polycyclic aromatic hydrocarbons or PAHs or Heavy metals or cadmium or Phytoestrogens or Polybrominated diphenyl ethers or PBDE or Polychlorinated biphenyls or PCBs or Dioxins or Triclosan) AND ((((Brain or Neuro) and (MRI or Diagnostic imaging or Imaging or Mapping or fMRI)) or Neuroimaging or Functional neuroimaging or Diffusion tensor Imaging) AND (fetus or fetal or prenatal or foetus or foetal or infant or preschool or postnatal or toddler or infant or child or children or childhood or pediatric or puber or puberty or adolescent or teenager or offspring or progeny))

1. **eMethods. Supplemental methods**

**Search strategy (methods)**

Two independent reviewers screened the titles and abstracts. Any disagreements between the two reviewers were resolved through the consultation of a third independent reviewer. Next, two independent reviewers screened the full article, reporting the reasons for exclusion. Any disagreements between the two reviewers were resolved through the consultation of a third independent reviewer. Finally, the references to the retrieved studies were scanned to identify additional relevant publications that were missed by the initial search strategy, resulting in four additional inclusions.

## Data extraction and quality assessment

Data extraction was performed by two independent reviewers, and a consensus was reached with the involvement of a third reviewer. A predesigned data abstraction tool was used to extract relevant information. For the quality assessment was used for cohort and case-control studies, the nine-star Newcastle-Ottawa Scale (NOS), which includes three predefined domains: selection of participants (population representativeness), comparability (adjustment for confounders), and ascertainment of outcome of interest. The NOS assigns a maximum of four points for selection, two points for comparability, and three points for outcome. A score of more than five reflects adequate quality for inclusion in the review, and nine points on the NOS reflect the highest quality of the study.

1. **sFigure 1. Flowchart of literature review**


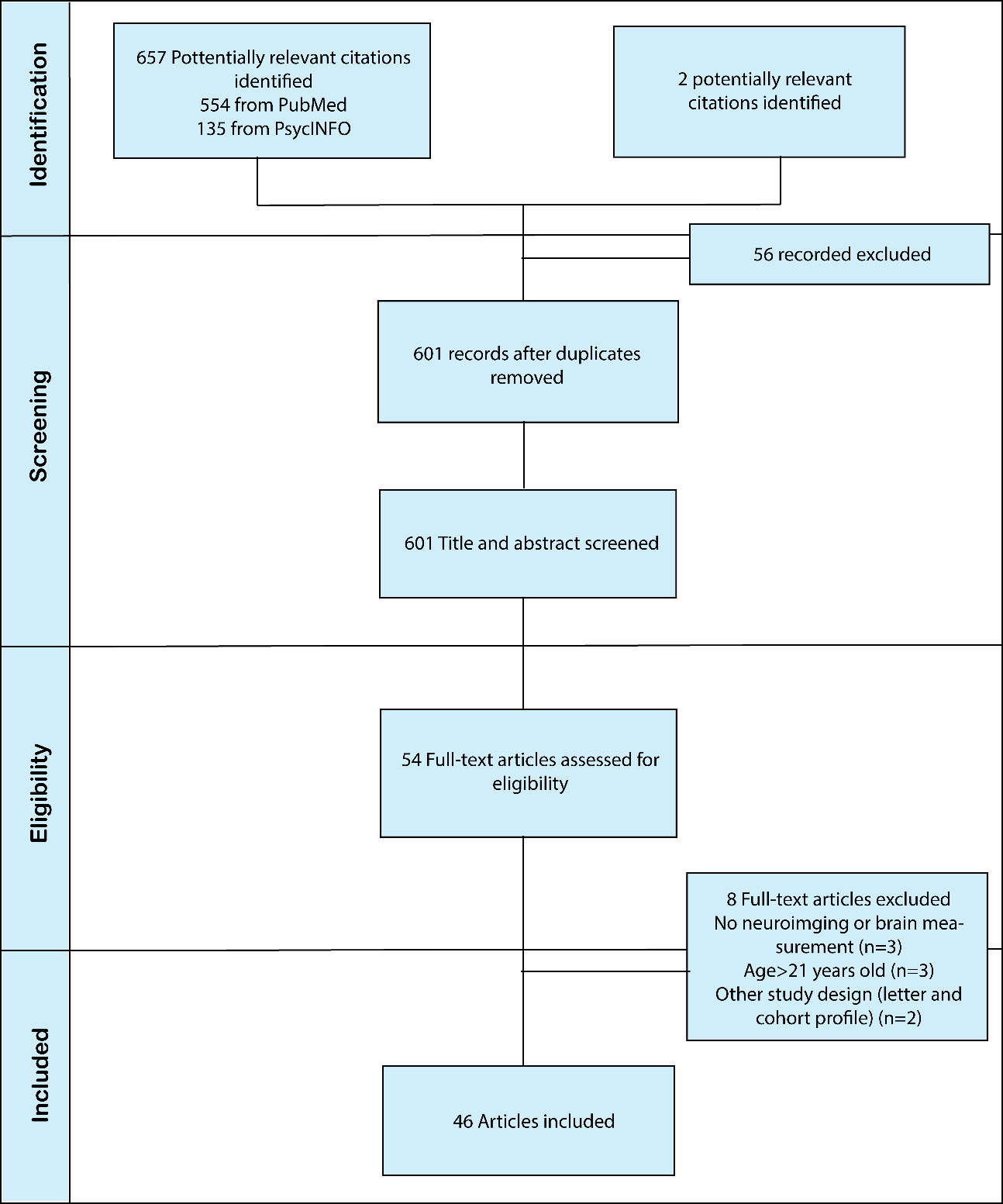

Supplement: Supplementary file 1 — (DOCX 115 KB) [file 40572_2024_457_MOESM1_ESM.docx]
